# Macular Thickness Profile and Its Association With Best-Corrected Visual Acuity in Healthy Young Adults

**DOI:** 10.1167/tvst.10.3.8

**Published:** 2021-03-10

**Authors:** Samantha Sze-Yee Lee, Gareth Lingham, David Alonso-Caneiro, Jason Charng, Fred Kuanfu Chen, Seyhan Yazar, David Anthony Mackey

**Affiliations:** 1 Centre for Ophthalmology and Visual Science (incorporating the Lions Eye Institute), University of Western Australia, Western Australia, Australia; 2 Queensland University of Technology, Contact Lens and Visual Optics Laboratory, Centre for Vision and Eye Research, School of Optometry and Vision Science, Queensland, Australia; 3 Royal Perth Hospital, Western Australia, Australia; 4 Single Cell and Computational Genomics Lab, Garvan Institute of Medical Research, New South Wales, Australia; 5 Centre for Eye Research Australia, University of Melbourne, Royal Victorian Eye and Ear Hospital, Victoria, Australia; 6 School of Medicine, Menzies Research Institute Tasmania, University of Tasmania, Tasmania, Australia

**Keywords:** retinal thickness, best-corrected visual acuity, the Raine Study, ganglion cell-inner plexiform layer, outer retinal layers

## Abstract

**Purpose:**

To describe the thickness profiles of the full retinal and outer retinal layers (ORL) at the macula in healthy young adults, and associations with best-corrected visual acuity (BCVA).

**Methods:**

In total, 1604 participants (19–30 years) underwent an eye examination that included measurements of their BCVA, axial length, and autorefraction. The retinal thickness at the foveal pit and at the nine Early Treatment of Diabetic Retinopathy Study macular regions (0.5-mm radius around the fovea, and superior, inferior, temporal, and nasal quadrants of the inner and outer rings of the macula) were obtained using spectral-domain optical coherence tomography imaging. A custom program was used to correct for transverse magnification effects because of different axial lengths.

**Results:**

The median full retinal and ORL thicknesses at the central macula were 285 µm and 92 µm. The full retina was thinnest centrally and thickest at the inner macula ring, whereas the ORL was thickest centrally and gradually decreased in thickness with increasing eccentricity. There was no association between axial length and the full retinal or ORL thickness. Increased thicknesses of the full retina at the central macula was associated with better BCVA; however, the effect size was small and not clinically significant.

**Conclusions:**

This article mapped the full retinal and ORL thickness profile in a population-based sample of young healthy adults.

**Translational Relevance:**

Thickness values presented in this article could be used as a normative reference for future studies on young adults and in clinical practice.

## Introduction

The retina is composed of highly metabolic tissue that is considered to be an extension of the brain. Changes in its morphology have been suggested to be a useful biomarker of some systemic,^1^ neurodegenerative,^2^ and ocular diseases.^3^ For example, the retinas of individuals with the autoimmune diseases systemic lupus erythematosus and Behcet's disease have been found to be thinner than those of healthy controls,^4^^,^^5^ even when there was no clinically obvious ocular involvement.^4^ Individuals with nonexudative age-related macular degeneration have been observed to have thinner retinas,^6^ whereas various forms of macular edema intrinsically result in thickening of the central retina. Myopic eyes, interestingly, tend to have thicker retinas at the central macula but thinner retinas at the inner and outer macula.^7^^–^^10^

Several studies have also reported associations between macular thickness and best-corrected visual acuity (BCVA) in eyes with macular pathology or high myopia. Other studies have reported that thinner foveas in eyes with macular edema after diabetes or after intraocular surgery correspond to better BCVAs.^11^^–^^13^ This association is reversed in other diseases such as nonexudative age-related macular degeneration^6^ and retinitis pigmentosa without macular cysts,^14^ with thicker retinas corresponding to better BCVA. Similarly, in individuals with high myopia, Flores-Morena et al.^15^ reported that BCVA was associated with thicker combined photoreceptor layer and pigmented retinal epithelium (the outer retinal layers) at the macula, but not with the foveal thickness. Recently, the Singapore Epidemiology of Eye Diseases study^16^ demonstrated that the association between BCVA and retinal thickness is present even in a general population of middle-aged and older adults with healthy eyes.

Despite the vital role of the retina in vision and the known changes in its morphology in disease, there has been surprisingly little information on the normal retinal thickness profile in young healthy eyes. Although researchers have mapped the retinal thickness in general populations of children^17^^–^^19^ and middle-aged or older adults,^16^^,^^20^ studies on young adults have been limited to those with myopia.^8^^,^^9^^,^^21^ Only one study^22^ described the retinal thickness at the macula in young healthy adults; however, its sample was relatively small, with the majority of participants having myopia (n = 124, 91% myopes). In this article, we describe the macular thickness profile of a large cohort of young and healthy individuals selected from the adults in a general population and its association with BCVA.

## Methods

### Study Sample

This study used data collected as part of the Kidskin Young Adult Myopia Study (KYAMS)^23^ and the Raine Study.^24^ Both studies had been approved by the Human Research Ethics Committee of the University of Western Australia and adhered to the tenets of the Declaration of Helsinki. All participants were given a full explanation of the nature of the study and provided written informed consent before participating.

The KYAMS is a follow-up of the Kidskin Study,^25^ which was a nonrandomized controlled trial in Western Australia in the late 1990s. The Kidskin Study investigated the value of an educational intervention on sun-protection habits in 5- to 6-year-old children. A total of 1776 children were recruited from primary (elementary) schools and assigned to a high-intervention, moderate-intervention, or a control group. The primary outcome measure was the longitudinal change in number of melanocytic nevi on the back, which was not significantly different between groups at the long-term follow-ups. All participants of the original Kidskin study were invited to the Lions Eye Institute in Perth, Western Australia, for an eye examination for the KYAMS, which took place between May 2015 and March 2019. The primary aim of the KYAMS was to explore associations between childhood sun-exposure habits and refractive outcomes in young adulthood.

The Raine Study is a cohort study that started in 1989 when 2900 pregnant women (termed “Gen1”) were recruited at 16 to 18 weeks’ gestation at the King Edward Memorial Hospital in Perth, Western Australia. Between November 1989 and March 1992, 2868 offspring (“Gen2”) were born to these women. Since then, these offspring have been undergoing a series of health and medical examinations. At the Gen2 20-year follow-up, participants attended an eye examination between March 2010 and February 2012 at the Lions Eye Institute.

Participants were excluded from the analysis if they reported a previous diagnosis of retinal or optic disc disease or there was an incidental finding of retinal or optic disc pathology during the eye examination. Participants with a history of any uveitis were also removed from the analysis given previous reports of decreased retinal thickness in autoimmune disease.^4^^,^^5^ Amblyopic eyes, defined as per previous studies^26^^–^^28^ (including in the Raine Study and the KYAMS cohorts),^29^^,^^30^ were additionally excluded as thicker retinas have been reported in amblyopic eyes.^31^^,^^32^ To avoid including participants with an unreported diagnosis of retinal or optic disc disease, eyes with BCVA <6/9 were further excluded from the analysis.

### Eye Examination

Presenting visual acuity (VA) was measured monocularly using logMAR-style charts, with participants wearing their habitual optical correction (if any) and scored letter-by-letter. For the KYAMS participants, an Early Treatment of Diabetic Retinopathy Study chart (ETDRS; Precision Vision, Woodstock, IL, USA) was used, while a Test Chart 2000 XPert (Thomson Software Solutions, Welham Green, UK) was used in the Raine Study. Participants were encouraged to read down the chart until no more than two letters could be identified correctly on a line. Regardless of the presenting VA, VA was also measured with pinholes on top of participants’ habitual distance correction. The presenting VA or the pinhole VA (with any habitual correction), whichever was better, was recorded as the BCVA.

Participants additionally underwent measurements of their axial length (IOLMaster v5; Carl Zeiss Meditec AC, Jena, Germany), central corneal thickness (CCT; Oculus Pentacam; Oculus Optikgerate GmbH, Wetzlar, Germany), and intraocular pressure (IOP; ICare TA01i; icare, Vantaa, Finland). Autorefraction and autokeratometry (Nidek ARK-510A Autorefractometer; Nidek Co Ltd, Tokyo, Japan) were performed at least 20 minutes after instillation of 1% tropicamide.

To obtain measures of the macular thicknesses, imaging of the posterior pole was performed using spectral-domain optical coherence tomography (SD-OCT; Spectralis HRA+OCT; Heidelberg Engineering, Heidelberg, Germany). Before SD-OCT imaging, the average autokeratometry value of each eye was entered into the imaging software to correct for ocular magnification effects. A 31-raster scan of a 30° × 25° area centered on the fovea was obtained from each eye, with each B-scan averaged from nine frames. Participants were informed to look at the fixation light, and the imaging examiner ensured that the macula was at the center of the scan area. All scans were taken with a default axial length of 24.385 mm and refractive error of 0 D, similar to the process in clinical practice on the Spectralis SD-OCT. Scan quality was maintained at a signal-to-noise ratio of 20 or higher^33^ and then assessed subjectively by the technician when the imaging was completed. Scans were repeated where necessary and if the participant was willing.

**Figure 1.**
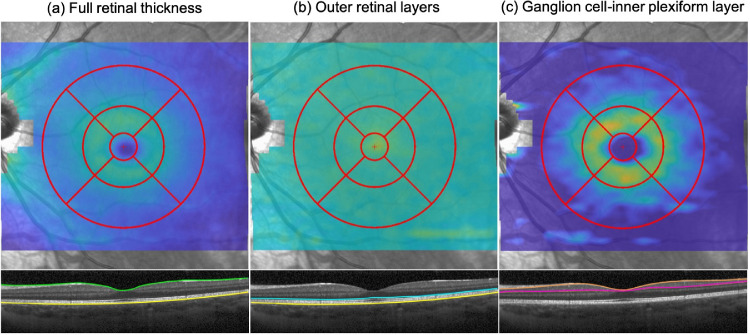
Layers segmentation by the custom program. (A) Full retinal thickness; (B) outer retinal layers (photoreceptors + retinal pigmented epithelium); (C) ganglion cell–inner plexiform layer.

The scans were exported and analyzed with a noncommercial custom program developed on MATLAB version R2017b (MathWorks, Inc. Natick, MA, USA). The program automatically measures the thickness of the full retinal layer, outer retinal layer (ORL; photoreceptor layer + retinal pigment epithelium), and ganglion cell–inner plexiform layers (GCIPL) in the nine regions defined by the ETDRS grid^34^ (Fig. 1): the central macula (0.5 mm radius around the fovea; C0), and the superior, temporal, inferior, and nasal quadrants of the inner ring (central region between 0.5 and 1.5 mm radius around the fovea; S1, T1, I1, and N1) and outer ring (central region between 1.5 and 3.0 mm radius around the fovea; S2, T2, I2, and N2) of the macula (Fig. 1). The program additionally corrects for lateral retinal image magnification effects induced by the different axial lengths. The main measures are the (1) minimum foveal thickness (fovea_min_; full thickness of the presumed foveal pit), and the thicknesses of the (2) full retinal, (3) and the ORL at the central macula. The fovea_min_ was automatically determined by the SD-OCT as the minimum thickness of the retina at the center ETDRS cell. Secondary measures included the full retinal and ORL at the rest of the eight macular regions. We additionally described the GCIPL at the inner and outer macula rings, but not at the central macula because of the absence of this layer in this region.

### Statistical Analysis

Statistical analyses were performed on the R Statistical Environment version 3.6.2 (The R Foundation for Statistical Programming, Vienna, Austria; https://www.r-project.org/). Continuous variables were expressed in terms of mean ± 1 SD or median and interquartile range (IQR) as appropriate. Cohort difference in age, sex, and ethnicity were determined using independent *t*-test for continuous variables and χ^2^ test for categorical variables.

We first identified ocular predictors of macular thickness measures in univariable models. Ocular measures that were significantly associated with macular thickness measures in the univariable models were included in the multivariable analyses. Because of the strong correlation between refractive error and axial length, only axial length was included as an independent measure in the analyses. Next, we explored the association of the three main measures (fovea_min_, full retinal, and ORL thicknesses) with BCVA as the dependent variable. All analyses involving ocular measures as the outcome were conducted using generalized estimating equations, because they were able to account for covariates, missing data, and the non-normal distribution of the data. To account for the within-subject correlation between two eyes, an exchangeable correlation structure was implemented in the models.^35^^,^^36^ The level of significance was set at *P* < 0.05. However, in the analyses involving macular thickness measures, this was adjusted to *P* < 0.017 with the Bonferroni correction applied to account for the multiple comparisons (0.05 divided by three main measures). To assess the impact of the different VA charts used in the KYAMS and Raine Study, we additionally performed a sensitivity analysis by exploring the relationship between BCVA and retinal thickness in the two cohorts separately.

## Results

**Figure 2.**
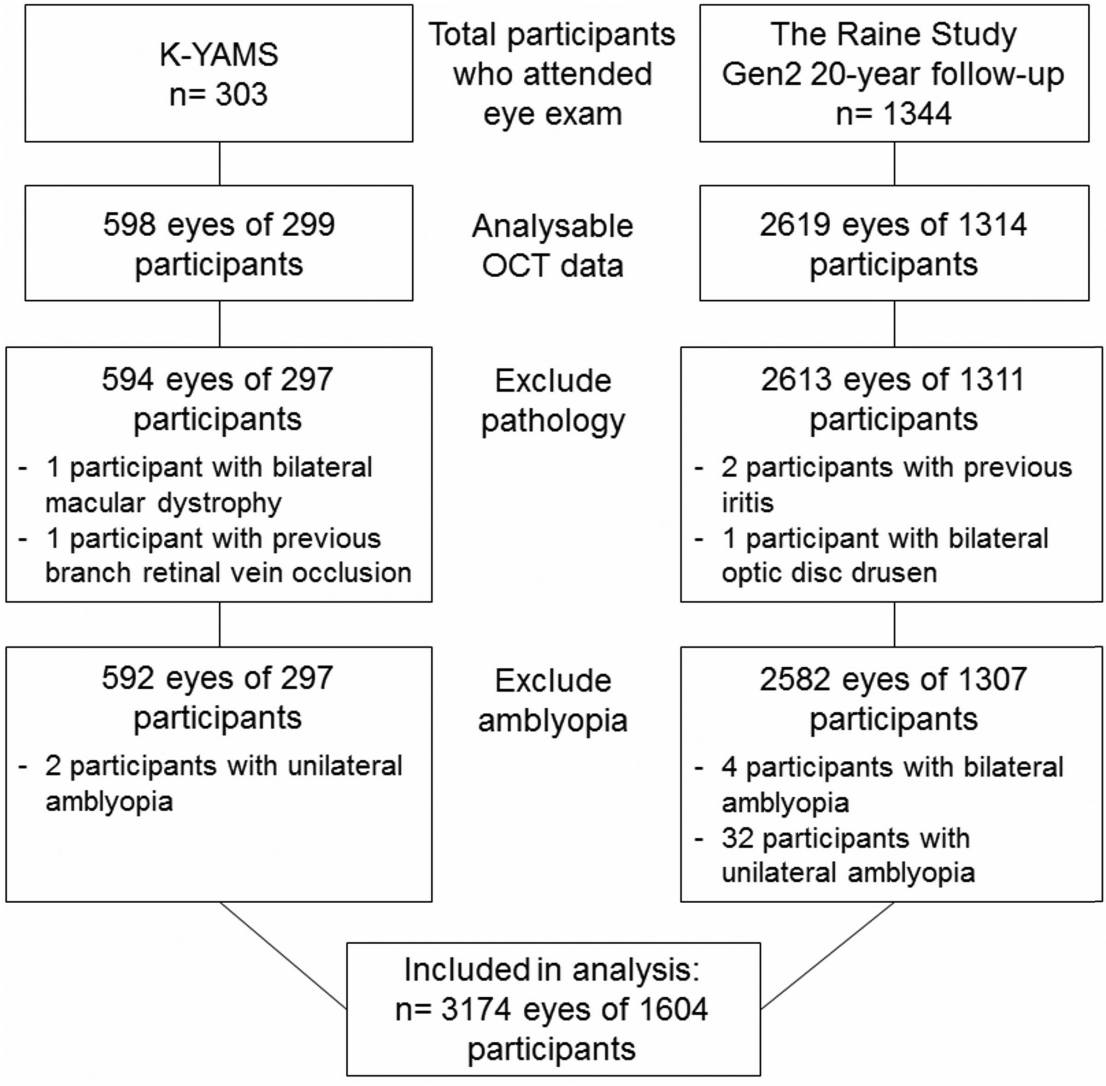
Study sample.

After excluding participants with poor SD-OCT scan qualities (including scans that are decentered, truncated, or with low signal-to-noise ratio), those with posterior segment pathology or previous uveitis, and amblyopic eyes, 3174 eyes of 1604 participants were included in the analysis (Fig. 2). There was no significant difference in age, sex, or ethnicity between those participants included and those removed from the analysis (*P* > 0.05). As shown in Table 1, the KYAMS Study participants were significantly older, with a higher proportion of females, than in the Raine Study. Additionally, the Raine Study participants were on average more hyperopic and had higher IOPs than the KYAMS cohort.

**Table 1.** Study Cohort Demographic and Ocular Measures

	Overall (n = 1604)	KYAMS (n = 297)	The Raine Study (n = 1307)	*P* Value^a^
Age^b^ yrs (mean ± SD)	21.1 ± 3.0	27.4 ± 1.1	20.1 ± 0.1	<0.001
Male sex^c^	784 (48.9%)	114 (38.4%)	670 (51.3%)	<0.001
Ethnicity^d^				0.39
Caucasian	1373 (85.6%)	256 (86.2%)	1117 (85.5%)	
East Asian	32 (2.0%)	3 (1.0%)	29 2.2%)	
South Asian	22 (1.4%)	2 (0.7%)	20 1.5%)	
Other/mixed	177 (11.0%)	36 (12.1%)	141 (10.8%)	
Ocular measures^e^ (median [IQR])				
BCVA	−0.08 [−0.10 to 0.00]	−0.10 [−0.12 to 0.00]	−0.06 [−0.10 to 0.00]	0.004
Spherical equivalent (D)	+0.25 [−0.38 to 0.63]	+0.00 [−0.63 to +0.50]	+0.25 [−0.38 to +0.63]	0.036
Axial length (mm)	23.5 [23.0 to 24.1]	23.5 [22.9 to 24.2]	23.5 [23.0 to 24.1]	0.28
Corneal radius (mm)	7.72 [7.56 to 7.90]	7.68 [7.54 to 7.89]	7.72 7.56 to 7.90]	0.11
CCT (µm)	538 [516 to 560]	535 [514 to 558]	539 [517 to 560]	0.06
IOP (mm Hg)	15 [13 to 17]	14 [11 to 16]	15 [13 to 18]	<0.001

BCVA, best-corrected visual acuity; CCT, central corneal thickness; IOP, intraocular pressure; IQR, interquartile range; KYAMS, Kidskin Young Adult Myopia Study; SD, standard deviation.

a Statistical significance set at *P* < 0.05.

b Cohort difference analyzed using independent sample *t*-test.

c Cohort difference analyzed using χ^2^ test.

d Cohort difference analyzed using Fisher's exact test.

e Cohort difference analyzed using generalized estimating equations.

### Macular Thickness Profile

**Figure 3.**
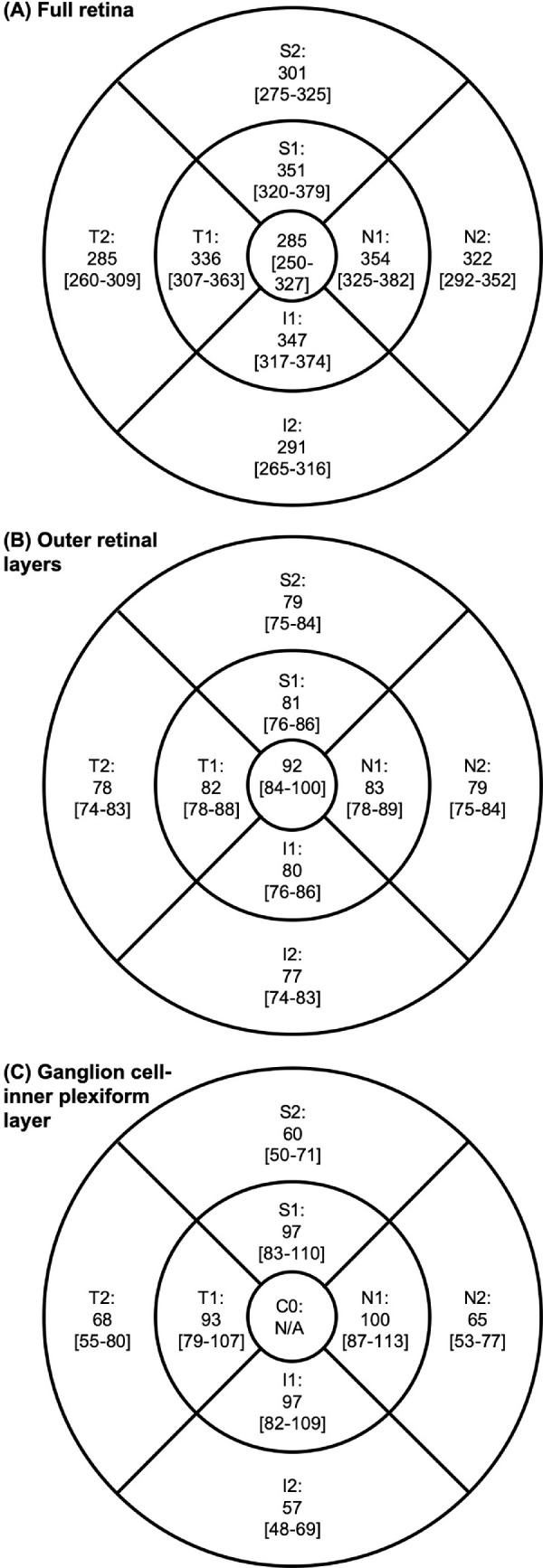
Median [and 2.5th to 97.5th percentile] thicknesses of the (**A**) full retina, (**B**) outer retinal layers, and (**C**) ganglion cell–inner plexiform layer (GCIPL) at the central macula (0.5 mm radius around the fovea), inner macular (region between 0.5 and 1.5 mm radius around the fovea; S1, T1, I1, and N1), and outer macula (regions between 1.5 and 3.0 mm radius around the fovea; S2, T2, I2, and N2). N/A, not applicable, as GCIPL not present at central macula.

The median fovea_min_ thickness of the combine cohort was 223 µm (95% confidence interval = 222 to 224; IQR= 214 to 235; 2.5th to 97.5th percentile = 198.0 to 269.1). The median thicknesses of the full retina, ORL, and GCIPL are shown in Figure 3 with the 2.5th and 97.5th percentile, and in Supplementary Figure S1 with the 95% confidence interval and IQR. The full retina was thickest at the N1, S1, and I1 regions of the macula, and thinnest centrally. The ORL, on the other hand, was thickest centrally and gradually thinned towards the periphery (Figs. 1 and 3). The median and 2.5th to 97.5th percentile of the full retinal, ORL, and GCIPL thicknesses broken down by sex and axial length are provided as Supplementary Material S2 to S5.

In the univariable analyses, corneal radius, CCT, and IOP were not significant predictors of any retinal thickness variable (*P* > 0.05) and were thus not included in the multivariable analyses. Table 2 shows the results of the multivariable analysis for predictors of the three main measures. Older age was independently associated with thinner ORL (*P* = 0.001), whereas all three macular thickness measures were significantly lower in females compared to males (all *P* < 0.001; Table 2). Additionally, Caucasians had thicker fovea_min_ and fuller retinas than those of other ethnicities (all *P* < 0.001).

**Table 2.** Multivariable Analyses for Predictors of the Main Retinal Thickness Measures (µm)

	Fovea_min_			Full Retina			ORL		
	Estimate [95%CI]	Wald χ^2^	*P* Value^a^	Estimate [95%CI]	Wald χ^2^	*P* Value^a^	Estimate [95%CI]	Wald χ^2^	*P* Value^a^
Age (per 1-year increase)	−0.28 [−1.7 to 1.1]	0.5	0.48	−1.8 [−3.4 to −0.3]	5.2	0.022	**−0.7 [−1.0 to −0.4]**	**19.4**	**<0.001**
Male sex (ref = female)	**5.9 [−42 to 7.7]**	**75.1**	**<0.001**	**11.0 [9.0 to 13.0]**	**120.9**	**<0.001**	**0.8 [0.4 to 1.2]**	**13.6**	**<0.001**
Ethnicity (ref = Caucasian)									
East Asian	**−6.0 [−11.3 to −0.6]**	**7.5**	**0.006**	**−12.8 [−19.7 to −5.8]**	**12.9**	**<0.001**	0.0 [−2.2 to 1.9]	0.0	0.98
South Asian	**−4.6 [−7.1 to −2.1]**	**30.2**	**<0.001**	**−12.4 [−19.1 to −5.6]**	**12.8**	**<0.001**	0.5 [−0.2 to 1.2]	1.7	0.19
Other/mixed	**−10.3 [−15.2 to −5.3]**	**22.7**	**<0.001**	**−10.9 [−14.0 to −7.8]**	**47.1**	**<0.001**	−0.5 [−2.3 to 1.4]	0.2	0.63
Raine study cohort (ref = KYAMS)	**−2.8 [−13.4 to 7.8]**	0.8	0.38	−11.8 [−23.5 to 0.0]	3.8	0.05	**−4.6 [−7.0 to −2.2]**	**14.0**	**<0.001**
Axial length (per 1 mm increase)	**1.3 [0.5 to 2.2]**	**17.1**	**<0.001**	−0.3 [−0.6 to 0.1]	1.7	0.19	−0.1 [−0.3 to −0.0]	2.4	0.12

Values in bold show significant associations.

CI, confidence interval; fovea_min_, minimum foveal thickness; KYAMS, Kidskin Young Adult Myopia Study; ORL, outer retinal layers.

a Statistically significant associations at *P* < 0.017 with the Bonferroni correction shown in bold.

Axial length was associated with the fovea_min_, which was reduced by 1.31 µm in thickness for each 1 mm increase in axial length. However, no relationship between axial length and the other retinal thickness measures at any macular regions were observed.

### Association With BCVA

**Figure 4.**
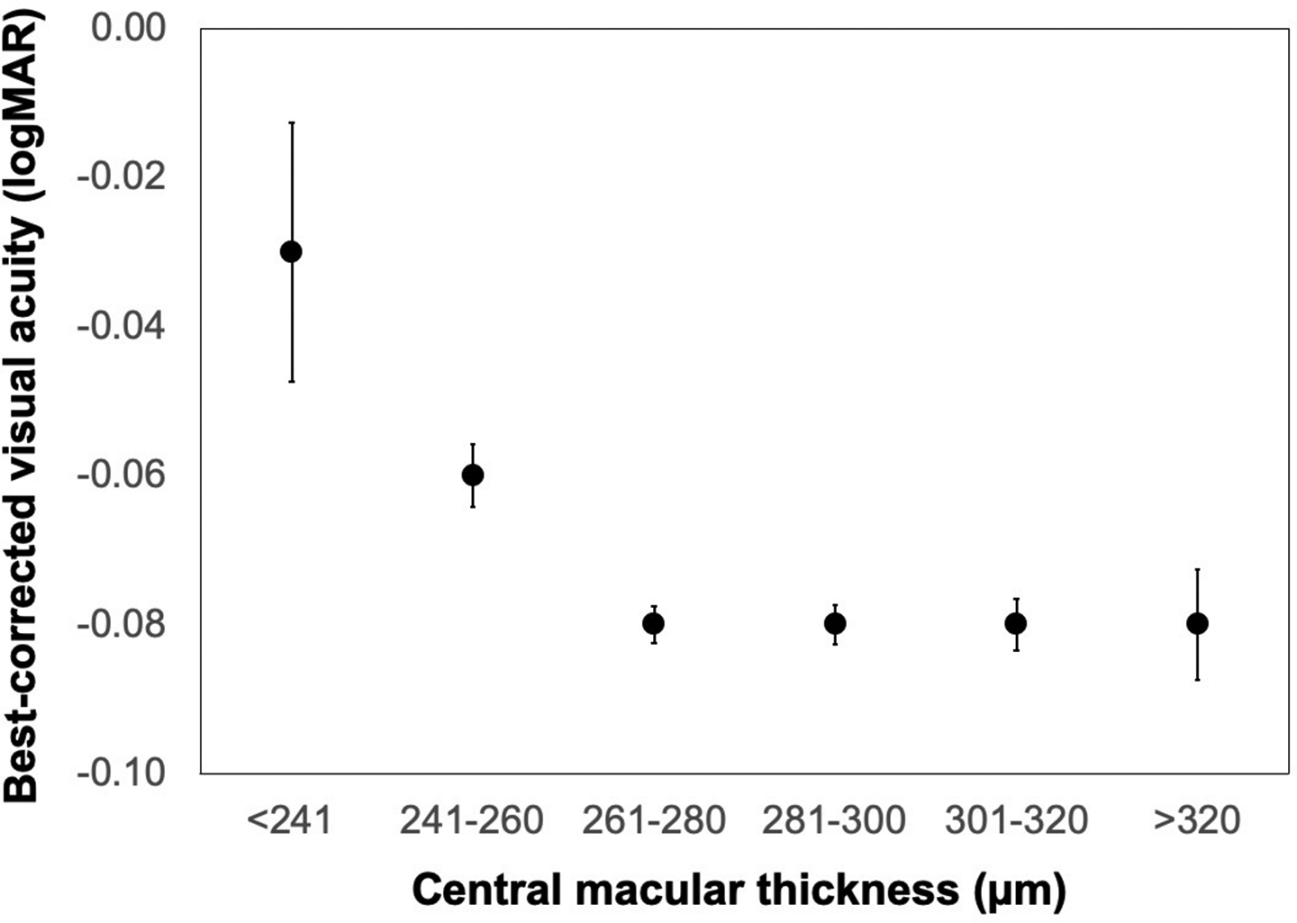
Best-corrected visual acuity as a function of full retinal thickness the central macula (0.5 mm radius around the fovea). *Error bars*: standard errors.

Thicker full retina at the central macula (C0 region) was independently associated with better BCVA (χ^2^ = 5.8, *P* = 0.016), after correcting for age, sex, ethnicity, cohort, and axial length. However, the effect size was small: an approximately 100 µm increase in full retinal thickness was associated with only a −0.06 logMAR change (a three-letter improvement) in BCVA (Fig. 4; Supplementary Fig. S6). Fovea_min_ and central ORL were not associated with BCVA (*P* = 0.16 and 0.12, respectively).

Increased thickness of the full retina at the I1, T1, and T2 regions of the macula were each independently associated with better BCVA (all *P* < 0.016). There was a trend toward similar associations between BCVA and the full retinal thickness at the other macular regions, as well as with the ORL thicknesses, but these did not reach statistical significance with the Bonferonni correction. In the sensitivity analysis, effect sizes and direction of association between BCVA and full retinal remained similar when the two cohorts were analyzed separately (Supplementary Table S7).

## Discussion

We described the macular thickness profile, including those of the presumed foveal pit, the full retina, ORL, and GCIPL, in a cohort of young healthy adults. In agreement with previous studies involving individuals of different ages and refractive status, the full retina^7^^–^^9^^,^^18^^–^^22^^,^^37^ was thickest superiorly, inferiorly, and nasally at the inner macular ring and thinnest centrally.

The full retinal thickness at the macula in young adults has been reported in earlier studies,^7^^–^^9^^,^^21^^,^^22^ but all included relatively small samples and individuals with myopia, and thus their findings may not be generalizable. In our study, we examined more than 1600 participants in a population-based sample of young Australian adults and found a full retinal thickness of 285 µm at the central macula, which is slightly thicker than values reported previously (187 to 255 µm) in smaller studies involving participants with low or no myopia.^7^^–^^9^ At the inner and outer macular rings, the full retinal thickness ranged from 285 to 354 µm in the current study, varying according to the ETDRS macular region, which was also thicker than those previously reported in young adults with low or no myopia (range 228 to 330 µm).^7^^–^^9^ There could be several reasons for this discrepancy. First, many of these previous studies were conducted in East Asian individuals who, as we have demonstrated, have thinner retinas compared to Caucasians, who were the majority of our sample. Vincent et al.^21^ similarly reported that their Caucasian participants had thicker retinas at most macular regions compared to individuals of Asian descent. Second, different SD-OCT models, which are known to have different definitions of the outer boundary of the retina, were used between studies. The Stratus identifies the outer boundary as the junction between the inner and outer segments of the photoreceptor layer, the Cirrus detects it just anteriorly to the interface of the RPE and the photoreceptors, while the Spectralis detects it at this interface. Correspondingly, the Spectralis, which was used in the current study, tends to produce higher thickness values compared to the Cirrus^38^ or Stratus,^39^ which were used in previous studies.^7^^–^^9^ Third, the macular measurements in our study were corrected for transverse magnification effects during the image analyses process. Although this is a more accurate measurement of the macular thickness in the ETDRS area, this may have not been done in most previous studies.^7^^–^^9^^,^^17^^,^^19^^,^^20^^,^^40^

The ORL and GCIPL thickness profiles in general populations have received limited attention in the literature. Using Cirrus high-definition OCTs, two studies in Asia^41^^,^^42^ and one in the United States^43^ reported average GCIPL thicknesses at the macula of 82 µm in participants aged 18 to 84 years. The authors additionally reported that sectoral macular GCIPL thickness ranged from 79 to 85 µm, with the thickest and thinnest sectors being the superonasal and inferotemporal, respectively. Unfortunately, we were unable to directly compare our findings with those of previous studies’ given the different regions of measurement at the macula between OCTs (i.e., Cirrus: 6 sectors versus Spectralis: 9 ETDRS regions). Nonetheless, we noted that the GCIPL was thickest at the nasal macular region, in accordance with previous studies.^41^^–^^43^ We also observed that the thickness profile of the GCIPL was similar to that of the full retina: thinnest at the central macular and thickest at the nasal, superior, and inferior inner macula.

In keeping with previous studies involving healthy children^17^ or adults of various ages,^44^^–^^46^ we found that the ORL was thickest at the central macula and evenly thinned in all directions away from the fovea. This thickness pattern reflects the longer outer segment of the cones and the taller retinal pigmented epithelial cells at and close to the fovea. The high density of photoreceptors at the fovea, which gradually decreases with increasing eccentricity, may also contribute to the ORL thickness pattern at the macula. We additionally found a significant inverse association between age and the three macular thickness measures, which reflects the decrease in photoreceptor density with increasing age.^47^ In their cross-sectional study of 297 adults aged 18 to 87 years, Nieves-Morena et al.^45^ reported that the ORL at the central macular decreases by 0.09 µm/yr, which is a considerably lower rate than our observed 0.6 µm/yr. However, the study by Nieves-Morena et al. ^45^ did not correct for potential confounders, such as sex and axial length. Moreover, given the cross-sectional nature of the previous and the current studies, any age effect should be interpreted conservatively until findings from longitudinal studies are available to draw further conclusions.

Previous reports^7^^–^^9^^,^^17^^,^^19^^,^^20^^,^^40^ have noted strong associations between axial length and full retinal or ORL thicknesses. Researchers have suggested that elongation of the eyeball in myopia occurs predominantly in the axial direction,^48^ which stretches out the retinal layers at the mid-periphery whereas the central retina is relatively less affected by the stretching. Sato et al.^49^ posited that traction of the vitreous with axial elongation results in elevation of the fovea, which may be linked to the increased the risk of macular pathologies in high myopia.^50^ However, with our magnification-corrected data, we failed to find such an association. Previous studies that have reported associations between axial length and retinal thickness did not correct for magnification effects. This would result in imaging over a larger area in longer eyes, leading to lower values of retinal thickness because of the SD-OCT software averaging the data points over larger and more peripheral areas. Likewise, the SD-OCT software would average the retinal thickness over a smaller central area in shorter eyes, consequently producing higher retinal thickness values.

We additionally found that the full retinal thickness was independently associated with BCVA. Indeed, several studies^6^^,^^11^^–^^14^^,^^51^^–^^54^ have reported that retinal thickness was an important predictor of VA in eyes with macular diseases, although the direction of association depended on the type of pathology. The Singapore Epidemiology of Eye Diseases study^16^ recently confirmed that the association between full retinal thickness and BCVA holds true even in older adults with healthy eyes, albeit with a small effect size.^16^ The effect size found in the current study was similarly small—with a 100 µm increase in central macular thickness (∼35% of the thickness) required for a three-letter improvement required for a one-letter improvement in BCVA. The relationship between retinal thickness and BCVA does not appear to be clinically significant.

A main strength of the current study is the large sample size of young adults in the general population with healthy eyes, resulting in a narrow 95% confidence interval. Although our participants were recruited from two cohort studies with statistically different ages and who were tested using different VA charts, we were able to demonstrate that the association between BCVA and retinal thickness was independent of cohort in the sensitivity analysis. Moreover, unlike most previous studies, we corrected for transverse magnification effects because of different axial lengths, which may increase the accuracy of the average retinal thickness measurements over the central ETDRS grid. A limitation of the study is the young age of our sample comprising a majority of Caucasians, and, hence, our findings may not be generalizable to other age groups or ethnicities. Additionally, we used a 31-line raster scan to map the macular thickness, which is less dense than that used in some studies.^13^^,^^16^^,^^21^ The macular thickness profile mapped by the current study may also not be suitable to be used as a reference in studies that use OCTs other than a Spectralis SD-OCT given the known discrepancy in retinal thickness measurements taken by different machines.^38^^,^^39^

In summary, we mapped the thickness profile of the full retina, ORL, and GCIPL at the macula in a population-based sample of healthy young adults, which serves as a useful reference for future studies on young Caucasian adults. Age and sex were key predictors of retinal thickness. Full retinal thickness is associated with BCVA; however, this association may not be clinically significant and varies according to macular region.

## Supplementary Material

Supplement 1

Supplement 2

Supplement 3

Supplement 4
