# Supplementary material for: Macular Thickness Profile and Its Association With Best-Corrected Visual Acuity in Healthy Young Adults
Source: Transl Vis Sci Technol. 2021 Mar 10;10(3):8. doi: 10.1167/tvst.10.3.8 (PMC7961121; doi:10.1167/tvst.10.3.8)
Supplement: Supplement 1 [file tvst-10-3-8_s001.pdf]

Supplementary material S2: minimum foveal thickness (presumed foveal pit; μm) in young Caucasian adults (19-30 years old), broken down by sex and axial length

	Male		Female	
**Axial length (mm)**	**Median**	**2.5^{th} - 97.5^{th} percentile**	**Median**	**2.5^{th} - 97.5^{th} percentile**
≤ 22.50	223.0	202.8 - 254.8	218.0	195.0 - 254.9
22.51 - 23.00	227.5	204.9 - 293.1	220.0	196.7 - 253.7
23.01 - 23.50	224.0	201.0 - 282.0	223.0	196.6 - 258.0
23.51 - 24.00	224.0	200.0 - 279.6	221.0	199.1 - 261.8
24.01 - 24.50	227.0	200.2 - 285.0	220.0	197.6 - 264.0
> 24.50	229.5	203.0 - 279.0	226.0	200.1 - 267.0

Supplementary material S3: Full retinal thickness (μm) at the macula in young Caucasian adults (19-30 years old), broken down by sex and axial length

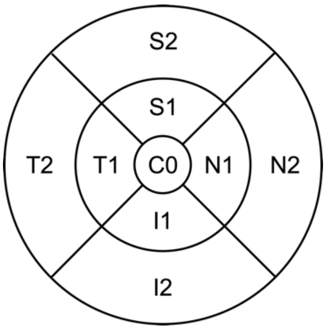

Macular regions: C0= central macula (0.5 mm radius around the fovea), inner macular (region between 0.5 and 1.5 mm radius around the fovea; S1, T1, I1, and N1), and outer macula (regions between 1.5 and 3.0 mm radius around the fovea; S2, T2, I2, and N2).

	**Male**		**Female**	
**Axial length (mm)**	**Median**	**2.5^{th} - 97.5^{th} percentile**	**Median**	**2.5^{th} - 97.5^{th} percentile**
Macular region C0				
≤ 22.50	291.1	255.7 - 330.1	279.8	245.7 - 322.4
22.51 - 23.00	294.9	255.7 - 334.5	280.4	243.9 - 315.1
23.01 - 23.50	291.0	255.4 - 333.6	280.3	247.1 - 319.2
23.51 - 24.00	289.7	253.6 - 330.5	279.1	250.1 - 318.1
24.01 - 24.50	289.3	256.9 - 326.6	278.0	249.0 - 310.9
> 24.50	289.7	254.1 - 329.4	277.5	236.8 - 316.0
Macular region S1				
≤ 22.50	356.7	326.0 - 379.9	347.2	321.8 - 368.7
22.51 - 23.00	355.1	327.9 - 380.7	347.7	313.7 - 370.3
23.01 - 23.50	357.1	330.0 - 383.6	347.8	316.9 - 373.3
23.51 - 24.00	354.2	328.3 - 383.4	347.3	318.0 - 372.5
24.01 - 24.50	353.5	331.5 - 377.7	345.9	317.5 - 373.7
> 24.50	354.7	325.9 - 379.6	343.5	318.3 - 370.6
Macular region S2				
≤ 22.50	303.8	276.9 - 331.6	297.8	274.2 - 323.8
22.51 - 23.00	300.7	278.2 - 323.4	299.3	275.1 - 322.5
23.01 - 23.50	302.6	276.4 - 328.1	299.9	272.5 - 323.5
23.51 - 24.00	302.2	277.5 - 325.2	299.3	275.6 - 325.2
24.01 - 24.50	302.1	276.8 - 323.7	300.1	278.6 - 327.5
> 24.50	303.2	270.1 - 326.3	297.3	274.2 - 319.6
Macular region I1				
≤ 22.50	353.9	323.8 - 376.8	339.1	320.0 - 369.7
22.51 - 23.00	352.1	319.6 - 375.5	344.0	316.3 - 369.2
23.01 - 23.50	353.9	328.7 - 380.2	342.6	314.6 - 371.5
23.51 - 24.00	351.5	322.8 - 376.5	343.3	314.5 - 370.2
24.01 - 24.50	350.1	322.6 - 373.3	341.9	314.4 - 365.3
> 24.50	352.1	322.4 - 374.3	338.8	312.9 - 365.6

Full retinal thickness (cont.)

	Male		Female	
**Axial length (mm)**	**Median**	**2.5th - 97.5th percentile**	**Median**	**2.5th - 97.5th percentile**
Macular region I2				
≤ 22.50	293.0	269.8 - 317.0	288.7	265.3 - 312.5
22.51 - 23.00	290.5	266.2 - 318.1	291.0	265.4 - 316.8
23.01 - 23.50	294.0	266.0 - 317.2	290.4	263.2 - 316.1
23.51 - 24.00	291.2	265.5 - 318.9	290.1	265.0 - 315.3
24.01 - 24.50	291.5	263.8 - 316.1	291.3	267.4 - 317.7
> 24.50	292.6	262.3 - 316.3	288.8	265.6 - 312.6
Macular region T1				
≤ 22.50	343.7	319.1 - 365.8	330.3	305.4 - 355.9
22.51 - 23.00	343.3	316.9 - 367.8	331.9	306.4 - 358.5
23.01 - 23.50	343.8	316.1 - 369.0	331.9	303.8 - 358.2
23.51 - 24.00	341.8	315.4 - 368.8	332.4	305.7 - 355.4
24.01 - 24.50	338.9	313.1 - 361.2	331.5	305.0 - 356.2
> 24.50	341.0	315.2 - 363.5	329.0	305.5 - 353.2
Macular region T2				
≤ 22.50	289.0	265.7 - 307.8	279.4	255.2 - 305.2
22.51 - 23.00	286.7	264.8 - 311.0	281.0	253.0 - 301.6
23.01 - 23.50	290.4	265.8 - 313.0	280.6	257.0 - 303.4
23.51 - 24.00	288.2	262.6 - 310.0	281.8	260.0 - 302.3
24.01 - 24.50	288.9	265.1 - 308.3	281.1	259.3 - 304.9
> 24.50	289.1	261.7 - 312.7	280.1	262.2 - 304.9
Macular region N1				
≤ 22.50	361.7	329.7 - 390.3	349.7	329.8 - 378.6
22.51 - 23.00	359.2	331.5 - 386.0	349.6	323.0 - 377.3
23.01 - 23.50	361.0	335.9 - 387.1	350.0	322.4 - 377.7
23.51 - 24.00	358.5	331.0 - 387.2	349.5	324.4 - 378.1
24.01 - 24.50	355.7	334.2 - 383.3	348.9	320.4 - 373.9
> 24.50	358.3	329.5 - 380.7	344.7	319.4 - 373.9
Macular region N2				
≤ 22.50	324.8	288.4 - 354.8	320.0	293.4 - 357.4
22.51 - 23.00	322.9	292.0 - 354.9	321.7	291.3 - 350.4
23.01 - 23.50	324.4	295.6 - 353.3	321.8	290.3 - 350.7
23.51 - 24.00	323.1	292.3 - 352.9	320.4	294.8 - 347.8
24.01 - 24.50	323.3	296.8 - 350.9	319.1	295.3 - 352.6
> 24.50	323.9	296.9 - 349.8	314.8	289.8 - 346.0

Supplementary material S4: Outer retinal layers thickness (μm) at the macula in young Caucasian adults (19-30 years old), broken down by sex and axial length

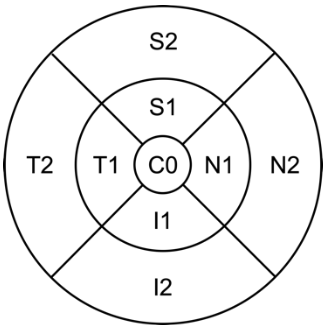

Macular regions: C0= central macula (0.5 mm radius around the fovea), inner macular (region between 0.5 and 1.5 mm radius around the fovea; S1, T1, I1, and N1), and outer macula (regions between 1.5 and 3.0 mm radius around the fovea; S2, T2, I2, and N2).

	**Male**		**Female**	
**Axial length (mm)**	**Median**	**2.5th - 97.5th percentile**	**Median**	**2.5th - 97.5th percentile**
Macular region C0				
≤ 22.50	93.2	84.3 - 99.0	91.9	84.3 - 100.1
22.51 - 23.00	91.6	83.8 - 99.6	1.1	84.0 - 99.5
23.01 - 23.50	92.6	83.4 - 100.4	91.9	84.3 - 100.3
23.51 - 24.00	92.6	82.7 - 101.6	91.0	83.0 - 99.2
24.01 - 24.50	102.0	90.9 - 112.9	91.0	83.2 - 99.8
> 24.50	91.7	84.2 - 99.2	91.2	83.2 - 98.5
Macular region S1				
≤ 22.50	81.2	76.5 - 86.1	81.0	76.6 - 85.4
22.51 - 23.00	81.7	76.7 - 85.2	80.5	76.4 - 86.1
23.01 - 23.50	81.6	77.1 - 86.6	81.3	76.5 - 86.8
23.51 - 24.00	81.4	77.0 - 86.5	81.2	76.1 - 85.3
24.01 - 24.50	81.1	77.0 - 86.0	80.7	75.2 - 86.1
> 24.50	81.5	76.8 - 86.8	80.4	76.1 - 86.4
Macular region S2				
≤ 22.50	79.5	74.8 - 82.8	78.8	74.6 - 83.8
22.51 - 23.00	79.2	76.0 - 83.0	78.2	74.5 - 83.8
23.01 - 23.50	79.4	75.4 - 84.6	78.9	74.4 - 83.8
23.51 - 24.00	79.2	75.5 - 83.9	79.1	74.4 - 83.3
24.01 - 24.50	79.3	75.3 - 83.7	78.8	73.9 - 83.1
> 24.50	79.5	75.4 - 84.9	78.4	74.8 - 82.9
Macular region I1				
≤ 22.50	80.2	76.1 - 84.1	80.0	75.5 - 86.8
22.51 - 23.00	81.0	76.3 - 86.1	79.7	75.5 - 85.6
23.01 - 23.50	81.1	76.0 - 86.9	80.4	76.0 - 85.1
23.51 - 24.00	80.6	76.6 - 86.1	80.1	75.8 - 85.6
24.01 - 24.50	80.3	76.4 - 85.7	79.9	76.2 - 85.0
> 24.50	81.0	76.9 - 87.2	79.7	75.5 - 84.5

Outer retinal layers thickness (cont.)

	Male		Female	
**Axial length (mm)**	**Median**	**2.5th - 97.5th percentile**	**Median**	**2.5th - 97.5th percentile**
Macular region I2				
≤ 22.50	77.2	74.1 - 81.1	76.5	73.3 - 81.8
22.51 - 23.00	77.2	73.9 - 82.7	76.4	73.4 - 82.2
23.01 - 23.50	77.3	74.1 - 82.6	76.7	73.6 - 82.6
23.51 - 24.00	77.4	73.9 - 82.9	77.1	73.6 - 82.5
24.01 - 24.50	77.6	73.8 - 82.0	76.7	73.4 - 82.0
> 24.50	77.8	74.3 - 83.2	76.9	73.5 - 80.7
Macular region T1				
≤ 22.50	82.8	78.2 - 88.7	82.0	77.0 - 89.3
22.51 - 23.00	82.5	78.4 - 88.1	81.5	76.7 - 86.9
23.01 - 23.50	83.0	78.1 - 88.5	82.4	77.0 - 88.1
23.51 - 24.00	82.7	77.5 - 89.3	82.1	76.9 - 87.7
24.01 - 24.50	82.5	77.4 - 87.1	82.0	76.4 - 87.6
> 24.50	82.6	78.4 - 87.7	81.9	77.5 - 87.5
Macular region T2				
≤ 22.50	77.9	74.5 - 81.9	77.6	74.1 - 83.3
22.51 - 23.00	77.9	74.5 - 81.8	77.3	73.5 - 82.2
23.01 - 23.50	78.2	73.8 - 82.7	77.8	73.8 - 82.5
23.51 - 24.00	78.0	74.1 - 82.8	77.8	74.0 - 81.6
24.01 - 24.50	77.7	74.0 - 82.0	77.8	73.6 - 81.3
> 24.50	78.3	74.4 - 83.3	77.2	74.0 - 81.4
Macular region N1				
≤ 22.50	83.7	78.5 - 88.8	82.7	77.0 - 87.9
22.51 - 23.00	83.3	79.5 - 88.2	82.6	77.8 - 88.3
23.01 - 23.50	83.8	78.3 - 88.9	83.5	78.1 - 89.6
23.51 - 24.00	83.6	78.7 - 89.3	83.0	77.8 - 88.5
24.01 - 24.50	83.6	78.7 - 88.2	83.3	77.8 - 88.5
> 24.50	83.3	79.1 - 88.6	82.7	77.8 - 88.0
Macular region N2				
≤ 22.50	78.9	74.3 - 83.2	78.1	74.1 - 83.1
22.51 - 23.00	79.3	74.9 - 84.1	78.0	74.2 - 83.3
23.01 - 23.50	79.4	75.0 - 84.6	78.8	74.8 - 83.7
23.51 - 24.00	79.2	74.9 - 84.0	78.8	74.2 - 83.8
24.01 - 24.50	79.3	75.0 - 83.2	78.6	74.6 - 82.7
> 24.50	79.8	75.4 - 84.9	78.3	74.2 - 83.2

Supplementary material S5: Ganglion cell + inner plexiform layers thickness (μm) at the macula in young Caucasian adults (19-30 years old), broken down by sex and axial length

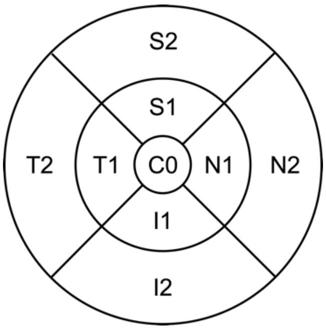

Macular regions: C0= central macula (0.5 mm radius around the fovea), inner macular (region between 0.5 and 1.5 mm radius around the fovea; S1, T1, I1, and N1), and outer macula (regions between 1.5 and 3.0 mm radius around the fovea; S2, T2, I2, and N2).

	**Male**		**Female**	
**Axial length (mm)**	**Median**	**2.5th - 97.5th percentile**	**Median**	**2.5th - 97.5th percentile**
Macular region C0#				
≤ 22.50	-	-	-	-
22.51 - 23.00	-	-	-	-
23.01 - 23.50	-	-	-	-
23.51 - 24.00	-	-	-	-
24.01 - 24.50	-	-	-	-
> 24.50	-	-	-	-
Macular region S1				
≤ 22.50	98.9	80.9 - 110.1	94.9	78.8 - 108.1
22.51 - 23.00	97.9	82.0 - 110.5	95.6	82.1 - 108.3
23.01 - 23.50	99.2	87.4 - 112.9	95.2	82.9 - 108.0
23.51 - 24.00	99.1	83.9 - 110.3	95.5	81.1 - 106.6
24.01 - 24.50	99.2	87.3 - 110.6	94.8	84.7 - 107.3
> 24.50	99.3	85.0 - 111.3	94.7	83.1 - 107.9
Macular region S2				
≤ 22.50	60.5	49.5 - 71.1	58.3	48.9 - 67.9
22.51 - 23.00	60.2	51.9 - 71.6	59.5	48.8 - 68.3
23.01 - 23.50	60.6	50.7 - 71.9	59.5	49.9 - 69.2
23.51 - 24.00	61.5	51.1 - 70.3	60.0	49.8 - 70.2
24.01 - 24.50	61.4	52.4 - 72.1	59.6	50.9 - 69.4
> 24.50	61.9	50.8 - 72.7	60.2	50.6 - 71.1
Macular region I1				
≤ 22.50	99.2	82.4 - 109.0	93.9	77.6 - 107.1
22.51 - 23.00	98.3	80.4 - 109.3	94.9	83.5 - 107.4
23.01 - 23.50	98.8	88.4 - 110.9	94.8	82.6 - 107.8
23.51 - 24.00	98.3	84.5 - 108.8	95.0	80.5 - 106.9
24.01 - 24.50	98.2	86.6 - 110.4	94.8	84.0 - 107.3
> 24.50	99.3	81.9 - 110.1	94.5	81.4 - 106.9

# GCIPL not present/measurable at central macular

Ganglion cell + inner plexiform layers thickness *(cont.)*

	**Male**		**Female**	
**Axial length (mm)**	**Median**	**2.5th - 97.5th percentile**	**Median**	**2.5th - 97.5th percentile**
Macular region I2				
≤ 22.50	56.7	46.4 - 70.6	55.8	44.8 - 65.8
22.51 - 23.00	57.0	47.0 - 71.5	57.2	47.6 - 67.4
23.01 - 23.50	57.8	49.2 - 70.0	56.8	48.4 - 68.5
23.51 - 24.00	57.5	49.1 - 68.3	57.1	47.7 - 67.7
24.01 - 24.50	58.1	49.9 - 67.6	57.3	49.4 - 67.0
> 24.50	59.3	48.0 - 70.0	57.6	48.0 - 69.9
Macular region T1				
≤ 22.50	95.6	79.5 - 106.5	90.2	75.6 - 103.5
22.51 - 23.00	95.4	80.1 - 107.9	91.3	77.0 - 106.0
23.01 - 23.50	96.8	82.7 - 109.2	91.4	76.7 - 105.1
23.51 - 24.00	95.9	81.1 - 108.0	91.4	76.3 - 104.5
24.01 - 24.50	96.3	83.9 - 106.6	91.0	79.7 - 104.5
> 24.50	95.3	81.7 - 107.0	90.9	78.4 - 103.4
Macular region T2				
≤ 22.50	68.3	55.4 - 81.9	64.5	49.9 - 77.7
22.51 - 23.00	68.0	54.3 - 82.1	66.1	54.9 - 76.5
23.01 - 23.50	68.9	57.0 - 80.8	65.9	56.0 - 77.0
23.51 - 24.00	69.4	55.4 - 78.8	66.8	55.9 - 77.3
24.01 - 24.50	69.8	58.6 - 80.3	66.4	56.6 - 80.5
> 24.50	69.6	54.2 - 83.7	67.4	56.5 - 80.5
Macular region N1				
≤ 22.50	102.9	86.7 - 112.6	98.3	84.1 - 110.0
22.51 - 23.00	101.2	86.5 - 112.3	98.6	88.1 - 112.7
23.01 - 23.50	103.3	91.0 - 114.7	98.0	86.9 - 110.7
23.51 - 24.00	101.8	86.2 - 113.1	97.9	86.8 - 110.3
24.01 - 24.50	102.0	90.9 - 112.9	97.7	86.5 - 112.5
> 24.50	101.7	89.1 - 112.1	96.7	85.8 - 111.0
Macular region N2				
≤ 22.50	64.4	52.5 - 78.7	61.7	51.3 - 74.7
22.51 - 23.00	64.0	53.5 - 79.2	63.5	54.5 - 74.5
23.01 - 23.50	64.9	54.2 - 75.9	64.5	53.9 - 76.2
23.51 - 24.00	65.7	51.5 - 75.7	64.1	54.3 - 75.4
24.01 - 24.50	66.7	56.9 - 78.4	64.2	54.7 - 73.9
> 24.50	66.6	54.8 - 79.0	64.8	54.6 - 76.6
