# Supplementary material for: Macular Thickness Profile and Its Association With Best-Corrected Visual Acuity in Healthy Young Adults
Source: Transl Vis Sci Technol. 2021 Mar 10;10(3):8. doi: 10.1167/tvst.10.3.8 (PMC7961121; doi:10.1167/tvst.10.3.8)
Supplement: Supplement 2 [file tvst-10-3-8_s002.pdf]

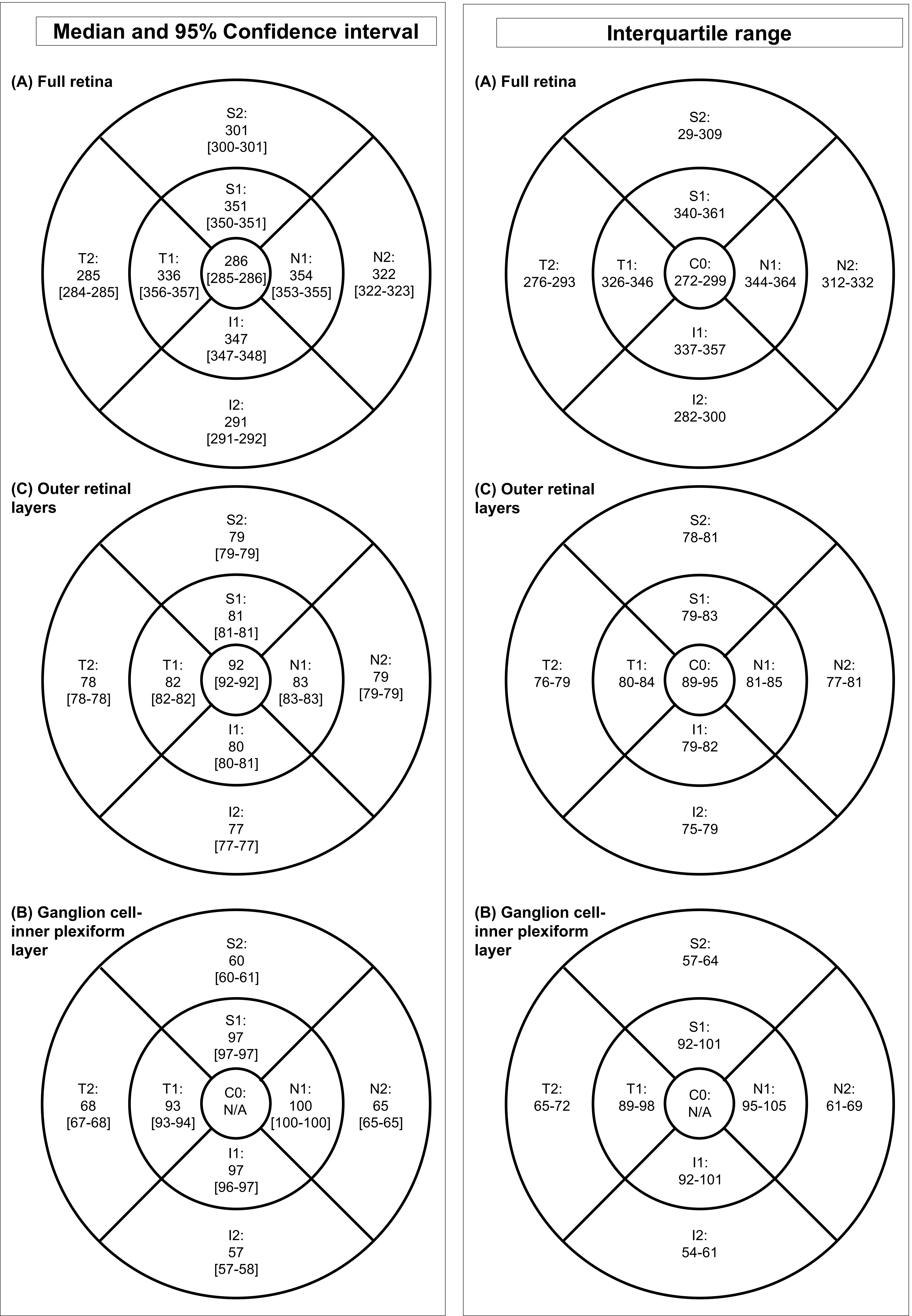


**Supplementary Figure S1**. Left: Median thicknesses and 95% confidence interval; and Right: Interquartile range of the: (a) full retina, (b) outer retinal layers, and (c) ganglion cell-inner plexiform layer at the at the central macula (0.5 mm radius around the fovea), inner macular (region between 0.5 and 1.5 mm radius around the fovea; S1, T1, I1, and N1), and outer macula (regions between 1.5 and 3.0 mm radius around the fovea; S2, T2, I2, and N2).
N/A= Not applicable
