# Supplementary material for: Macular Thickness Profile and Its Association With Best-Corrected Visual Acuity in Healthy Young Adults
Source: Transl Vis Sci Technol. 2021 Mar 10;10(3):8. doi: 10.1167/tvst.10.3.8 (PMC7961121; doi:10.1167/tvst.10.3.8)
Supplement: Supplement 3 [file tvst-10-3-8_s003.pdf]

Supplementary Table S7. Association of best-corrected visual acuity per 100μm increased in full retinal thickness and per 20 μm increased in outer retinal layers thickness at the macula

Macular region	All participants (n=1604)			KYAMS (n= 297)			Raine Study (n=1307)		
	**Estimate [95%CI]**	**Wald χ^2**	**p-value***	**Estimate [95%CI]**	**Wald χ^2**	**p-value***	**Estimate [95%CI]**	**Wald χ^2**	**p-value***
**Full retinal thickness** (per 100μm increase)									
C0	**-0.02 [-0.04 to 0.00]**	**5.8**	**0.016**	-0.00 [-0.04 to 0.03]	0.0	0.91	-0.00 [-0.04 to 0.03]	0.0	0.91
S1	-0.02 [-0.05 to 0.00]	3.9	0.05	-0.00 [-0.05 to 0.05]	0.0	0.89	-0.00 [-0.05 to 0.05]	0.0	0.89
S2	-0.02 [-0.05 to 0.00]	3.3	0.07	-0.02 [-0.07 to 0.04]	0.4	0.51	-0.02 [-0.07 to 0.04]	0.4	0.51
I1	**-0.03 [-0.05 to -0.01]**	**6.3**	**0.012**	-0.00 [-0.05 to 0.05]	0.0	0.99	-0.00 [-0.05 to 0.05]	0.0	0.99
I2	-0.02 [-0.04 to 0.01]	1.7	0.19	-0.01 [-0.05 to 0.04]	0.0	0.83	-0.01 [-0.05 to 0.04]	0.0	0.83
T1	**-0.03 [-0.06 to -0.01]**	**6.0**	**0.014**	-0.01 [-0.06 to 0.05]	0.1	0.80	-0.01 [-0.06 to 0.05]	0.1	0.80
T2	**-0.03 [-0.06 to -0.01]**	**7.1**	**0.008**	-0.00 [-0.06 to 0.06]	0.0	0.97	-0.00 [-0.06 to 0.06]	0.0	0.97
N1	-0.02 [-0.04 to 0.00]	3.2	0.07	-0.01 [-0.06 to 0.04]	0.1	0.73	-0.01 [-0.06 to 0.04]	0.1	0.73
N2	-0.01 [-0.03 to 0.01]	0.8	0.38	-0.02 [-0.06 to 0.03]	0.0	0.54	-0.02 [-0.06 to 0.03]	0.4	0.54
**Outer retinal layers thickness** (per 100 μm increase)									
C0	-0.06 [-0.14 to 0.02]	2.5	0.117	-0.02 [-0.26 to 0.22]	0.0	0.89	-0.07 [-0.15 to 0.01]	2.7	0.10
S1	-0.11 [-0.25 to 0.03]	2.3	0.128	-0.02 [-0.44 to 0.40]	0.0	0.93	-0.13 [-0.27 to 0.02]	2.8	0.09
S2	-0.14 [-0.29 to 0.00]	3.8	0.053	-0.10 [-0.48 to 0.29]	0.2	0.62	-0.15 [-0.31 to 0.00]	3.7	0.06
I1	-0.07 [-0.20 to 0.07]	1.0	0.326	-0.04 [-0.39 to 0.30]	0.1	0.80	-0.07 [-0.21 to 0.07]	0.9	0.34
I2	-0.07 [-0.22 to 0.07]	1.0	0.319	-0.20 [-0.50 to 0.10]	1.7	0.19	-0.04 [-0.21 to 0.12]	0.3	0.61
T1	-0.13 [-0.25 to -0.01]	4.5	0.033	-0.15 [-0.53 to 0.24]	0.6	0.45	-0.13 [-0.25 to 0.00]	4.0	0.05

T2	-0.16 [-0.30 to -0.01]	4.4	0.037	-0.07 [-0.45 to 0.30]	0.1	0.71	-0.17 [-0.33 to -0.01]	4.5	0.03
N1	-0.09 [-0.21 to 0.03]	2.1	0.145	-0.03 [-0.42 to 0.36]	0.0	0.88	-0.10 [-0.23 to 0.03]	2.1	0.15
N2	-0.10 [-0.23 to 0.03]	2.1	0.144	-0.16 [-0.45 to 0.13]	1.1	0.29	-0.08 [-0.22 to 0.07]	1.0	0.31

Corrected for age, sex, ethnicity, and axial length. CI= confidence interval; KYAMS= Kidskin young adult myopia study. *p<0.017 taken as statistical significance. Significant associations in bold.
