# Supplementary material for: Macular Thickness Profile and Its Association With Best-Corrected Visual Acuity in Healthy Young Adults
Source: Transl Vis Sci Technol. 2021 Mar 10;10(3):8. doi: 10.1167/tvst.10.3.8 (PMC7961121; doi:10.1167/tvst.10.3.8)

Supplementary Figure S6: Best-corrected visual acuity as a function of the full retinal (top) and outer retinal layers (bottom) thicknesses at the central macular (0.5mm radius around the fovea). KYAMS= Kidskin Young Adults Myopia Study; logMAR= logarithm of the minimal angle of resolution

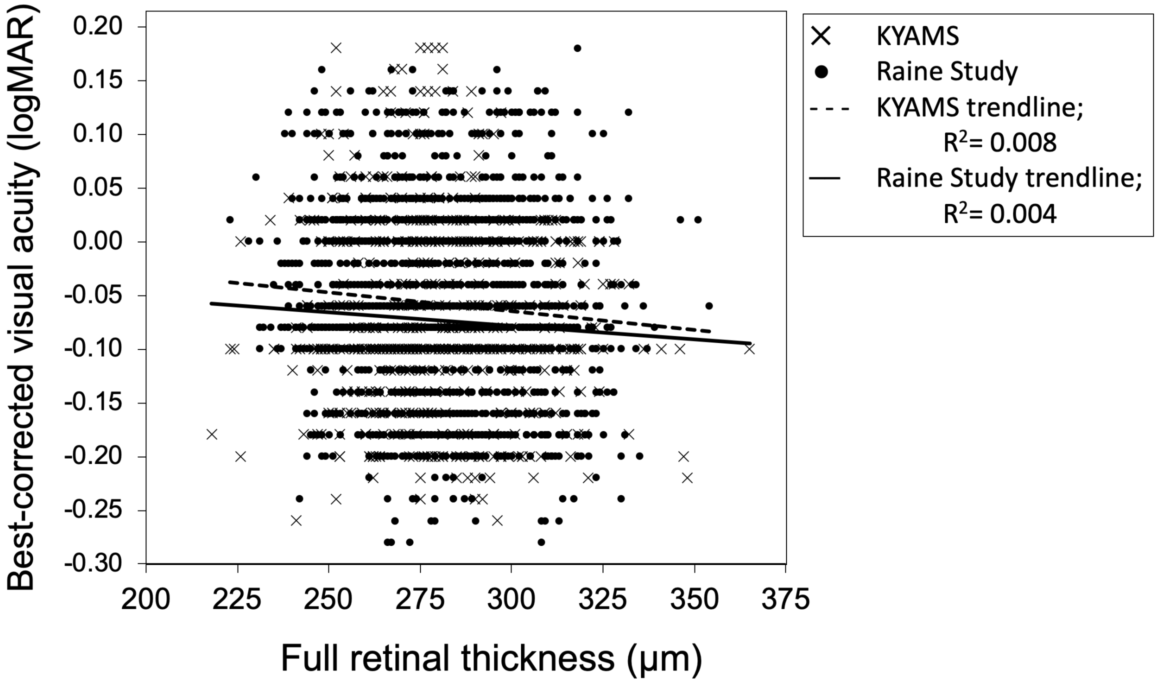

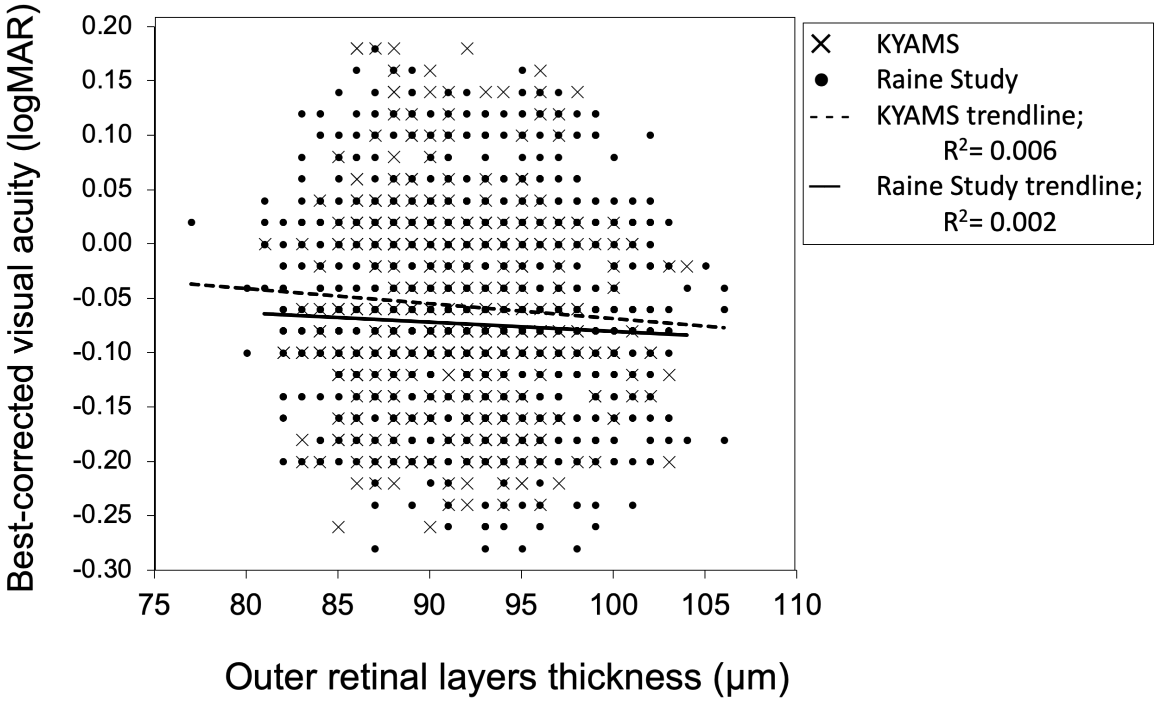

Supplement: Supplement 4 [file tvst-10-3-8_s004.pdf]
